# Gene Expression-Based Signature Can Predict Sorafenib Response in Kidney Cancer

**DOI:** 10.3389/fmolb.2022.753318

**Published:** 2022-03-14

**Authors:** Alexander Gudkov, Valery Shirokorad, Kirill Kashintsev, Dmitriy Sokov, Daniil Nikitin, Andrey Anisenko, Nicolas Borisov, Marina Sekacheva, Nurshat Gaifullin, Andrew Garazha, Maria Suntsova, Elena Koroleva, Anton Buzdin, Maksim Sorokin

**Affiliations:** ^1^ I. M. Sechenov First Moscow State Medical University, Moscow, Russia; ^2^ Moscow City Oncological Hospital №. 62, Moscow, Russia; ^3^ Moscow City Clinical Oncological Dispensary №. 1, Moscow, Russia; ^4^ Oncobox Ltd., Moscow, Russia; ^5^ Moscow Institute of Physics and Technology, Moscow, Russia; ^6^ World-Class Research Center “Digital Biodesign and Personalized Healthcare”, Sechenov First Moscow State Medical University, Moscow, Russia; ^7^ Department of Pathology, Faculty of Medicine, Lomonosov Moscow State University, Moscow, Russia; ^8^ OmicsWay Corp, Walnut, CA, United States; ^9^ Shemyakin-Ovchinnikov Institute of Bioorganic Chemistry, Moscow, Russia; ^10^ European Organization for Research and Treatment of Cancer (EORTC), Biostatistics and Bioinformatics Subgroup, Brussels, Belgium

**Keywords:** renal cell carcinoma, kidney cancer, gene signature, mRNA expression, RNA sequencing, microarray profiling, sorafenib response, tyrosine kinase inhibitor

## Abstract

Sorafenib is a tyrosine kinase inhibitory drug with multiple molecular specificities that is approved for clinical use in second-line treatments of metastatic and advanced renal cell carcinomas (RCCs). However, only 10–40% of RCC patients respond on sorafenib-containing therapies, and personalization of its prescription may help in finding an adequate balance of clinical efficiency, cost-effectiveness, and side effects. We investigated whether expression levels of known molecular targets of sorafenib in RCC can serve as prognostic biomarker of treatment response. We used Illumina microarrays to profile RNA expression in pre-treatment formalin-fixed paraffin-embedded (FFPE) samples of 22 metastatic or advanced RCC cases with known responses on next-line sorafenib monotherapy. Among them, nine patients showed partial response (PR), three patients—stable disease (SD), and 10 patients—progressive disease (PD) according to Response Evaluation Criteria In Solid Tumors (RECIST) criteria. We then classified PR + SD patients as “responders” and PD patients as “poor responders”. We found that gene signature including eight sorafenib target genes was congruent with the drug response characteristics and enabled high-quality separation of the responders and poor responders [area under a receiver operating characteristic curve (AUC) 0.89]. We validated these findings on another set of 13 experimental annotated FFPE RCC samples (for 2 PR, 1 SD, and 10 PD patients) that were profiled by RNA sequencing and observed AUC 0.97 for 8-gene signature as the response classifier. We further validated these results in a series of qRT-PCR experiments on the third experimental set of 12 annotated RCC biosamples (for 4 PR, 3 SD, and 5 PD patients), where 8-gene signature showed AUC 0.83.

## Introduction

According to the estimates for 2020, globally there were ∼431,000 new cases of kidney cancer and ∼179,000 associated deaths (Sung et al., 2021). Renal cell carcinoma (RCC) is the most common subtype of kidney cancer in adults, responsible for nearly 90% of all cases and prone to distant metastasis (He et al., 2021). RCC arises from the renal parenchyma, and the incidence of RCC is still increasing in most countries (Bhatt and Finelli, 2014; Du et al., 2020). Approximately 25–30% of RCC patients are diagnosed at a metastatic or locally advanced disease stage, and another third of RCC patients will recur after receiving a successful first-line treatment (Sánchez-Gastaldo et al., 2017). RCC includes several different histological subtypes with distinct biological behaviors and prognoses.

RCCs are frequently characterized by inactivation of the *VHL* tumor suppressor gene. This leads to deficiency of its encoded protein, which is part of an E3 ubiquitin ligase complex that degrades alpha subunit of hypoxia inducible factor 2 (HIF-2α). The resulting excessive accumulation of HIF-2α can transcriptionally upregulate oncogenic hypoxia-responsive genes, including platelet-derived growth factor (PDGF) and vascular endothelial growth factor (VEGF) (Choueiri and Kaelin, 2020). In turn, VEGF and PDGF promote angiogenesis, cell growth and survival, and RCC progression by activating the respective tyrosine kinase receptors PDGFR and VEGFR. This leads to high vascularization of RCC and to its high metastatic potential (He et al., 2021). Patients with metastatic RCC are insensitive to chemotherapy and radiotherapy, and have a poor survival prognosis (Choueiri and Motzer, 2017).

Sorafenib is a tyrosine kinase inhibitor with multiple specificities that targets at least ten tyrosine kinase molecules: RAF1, BRAF, RET, FLT1, FGFR1, KIT, PDGFRB, FLT3, FLT4, and KDR (Adnane et al., 2006). Sorafenib is thought to have a dual suppressive effect on tumors by blocking both angiogenesis, and cell proliferation and survival through the inhibition of VEGFR/PDGFR and BRAF/RET/FLT/FGFR/KIT/KDR signaling axis, respectively (Wilhelm et al., 2004, 2006). It is the first targeted drug approved for treatment of metastatic or locally advanced RCC by US FDA, which revolutionized treatment of kidney cancer and accelerated development and registration of other targeted therapeutics (Escudier et al., 2019). Since then, several other specific agents against VEGF, PDGF, and their receptors have been approved for the treatment of RCC, including sunitinib, axitinib, cabozanitinib, lenvatinib, pazopanib, and bevacizumab (Dizman et al., 2020). In addition, mTOR-specific inhibitors temsirolimus and everolimus were also approved for the treatment of this disease (Dizman et al., 2020). According to the National Comprehensive Cancer Network (NCCN) guidelines, sorafenib and sunitinib are recommended as drugs for metastatic RCC, where sorafenib has a lower toxicity than sunitinib (Deng et al., 2019). Moreover, sorafenib is also approved as the first-line treatment of metastatic RCC according to the latest guidelines of Chinese Society of Clinical Oncology (He et al., 2021). In clinical practice, it is also effective in hepatocellular and thyroid cancers (Escudier et al., 2019), myeloid leukemia, mesothelioma, and prostate cancer (Méndez-Blanco et al., 2018).

However, only 10–40% of RCC patients respond on sorafenib-containing therapeutic schemes (Larkin and Eisen, 2006; Escudier et al., 2007; Guevremont et al., 2009), and personalization of its prescriptions may help in finding an adequate balance of clinical efficiency, cost-effectiveness, and side effects. Nowadays, there are no clinical biomarkers of response on sorafenib treatment in RCC, and the molecular mechanisms of sorafenib resistance in RCC are not sufficiently understood (He et al., 2021). Currently, several RCC sorafenib resistance biomarkers were identified by RNA expression assays in drug responder and non-responder tumors: long non-coding RNAs *GAS5* (Liu et al., 2019) and *SRLR* (Xu et al., 2017), microRNA *miR-21*, and genes *ANGPTL3* (Bao et al., 2018), *CXCR4*, *CD34* (Aziz et al., 2014), *FGFR1* (Ho et al., 2015), *FRS2A*, *GLUT1*, *HO-1* (Zheng et al., 2015), *SOX5*, and *SOX9* (Li et al., 2015; He et al., 2021). In addition, expression of AKT protein was reported to be a biomarker of enhanced resistance against sorafenib in RCC patients (Jonasch et al., 2010). However, despite those important observations, no diagnostic settings were constructed to predict effectiveness of sorafenib for RCC patients.

Drug target expression levels, e.g., determined by immunohistochemistry, are already used as the biomarkers of treatment response in some solid cancers (Hechtman et al., 2017) including breast cancer (Nicolini et al., 2018) and gastric cancer (Abrahao-Machado and Scapulatempo-Neto, 2016). In addition, profiling of gene expression at RNA level is a powerful tool for discovery of drug efficiency biomarkers and for cancer therapy personalization (Buzdin et al., 2019). Previously, we showed that gene expression levels established from standardized RNA sequencing data can be used as robust estimators of the corresponding protein levels for several cancer biomarkers in tumor biosamples, including formalin-fixed paraffin-embedded (FFPE) specimens (Sorokin et al., 2020b).

In this study, we investigated FFPE biosamples of pre-treatment RCC tissues from 47 patients with known response status on next-line monotherapy with sorafenib. Illumina microarrays were used to profile RNA expression in FFPE samples of 22 metastatic or advanced RCC cases. Among them, nine patients showed partial response (PR), three patients—stable disease (SD), and 10 patients—progressive disease (PD) according to RECIST criteria. We then classified PR + SD patients as “responders” and PD patients as “poor responders”. We found that gene signature including eight sorafenib target genes was congruent with the drug response characteristics, and enabled high-quality separation of the responders and poor responders [area under a receiver operating characteristic curve (AUC) 0.89]. We validated these findings on another set of 13 experimental annotated FFPE RCC samples (for 2 PR, 1 SD, and 10 PD patients) that were profiled by RNA sequencing and observed AUC 0.97 for 8-gene signature as the response classifier. We further validated these results in a series of qRT-PCR experiments on the third experimental set of 12 annotated RCC biosamples (for 4 PR, 3 SD, and 5 PD patients), where 8-gene signature showed AUC 0.83.

## Materials and Methods

### Patients and Samples

All patients enrolled in this study have previously signed written informed consents to participate in the observational clinical investigation, and for publication of depersonalized molecular profiles and study results in the form of gene activity profiles associated with age, sex, and results of sorafenib monotherapy treatment estimated according to RECIST criteria (Eisenhauer et al., 2009). The patients provided written informed consent that their tumor samples will be subjected to gene expression profiling using either microarray technology or next-generation sequencing or qRT-PCR. Twenty-two patients signed agreement that their biosamples are profiled with the Illumina HT-12 bead arrays (Table 1). Thirteen patients signed agreement that their biosamples are profiled by RNA sequencing using Illumina HiSeq3000 next generation sequencing platform (Table 2). Twelve patients signed agreement that their biosamples are profiled by qRT-PCR, but not using expression microarrays or RNA sequencing (Table 3).

**TABLE 1:** Clinical information for RCC patients profiled using Illumina HumanHT-12 WG-DASL V4.0 R2 gene expression arrays

Patient ID	Response status	Age	Gender	T	N	M	Grade
18	Partial response	66	Male	2	0	1	4
26	Stable disease	64	Female	3	2	1	4
27	Progressive disease	53	Male	3	0	1	4
31	Partial response	62	Male	2	2	0	3
36	Progressive disease	60	Male	2	0	0	2
37	Partial response	49	Female	3	1	1	4
46	Progressive disease	45	Male	3	0	0	3
49	Progressive disease	66	Female	3	0	0	3
54	Partial response	55	Female	2	0	0	2
58	Progressive disease	65	Female	3	1	0	3
60	Progressive disease	59	Male	2	0	0	2
62	Progressive disease	58	Male	1	0	0	1
72	Partial response	56	Female	3	1	0	3
73	Progressive disease	48	Male	3с	0	0	3
74	Partial response	53	Male	4	2	1	4
88	Stable disease	59	Female	3	0	0	3
91	Stable disease	67	Female	3	2	1	4
94	Progressive disease	74	Female	3	1	1	4
97	Partial response	70	Female	3	0	0	3
122	Partial response	61	Male	3а	0	0	3
128	Partial response	68	Female	3	0	1	4
135	Progressive disease	50	Male	3	0	0	3

**TABLE 2:** Clinical information for RCC patients profiled using Illumina HiSeq3000 next-generation sequencing platform in this study

Patient ID	Response	Age	Gender	T	N	M	Grade
KC11	Progressive disease	62	Female	3	1	1	4
KC14	Progressive disease	68	Female	3	0	1	4
KC19	Partial response	46	Female	3	0	0	3
KC21	Progressive disease	41	Male	3	0	1	4
KC23	Progressive disease	53	Male	3	0	1	4
KC26	Stable disease	55	Male	3	0	1	4
KC36	Progressive disease	64	Female	3a	0	1	4
KC37	Partial response	54	Male	3a	0	1	4
KC46	Progressive disease	55	Male	3b	2	1	4
KC57	Progressive disease	58	Male	3b	0	1	4
KC92	Progressive disease	55	Male	3	0	0	3
KC93	Progressive disease	65	Female	2	0	0	2
KC96	Progressive disease	47	Male	3	0	0	3

**TABLE 3:** Outline of clinical information of patients whose samples were profiled using RT-PCR platform in this study

Patient ID	Response	Age	Gender	T	N	M	Grade
III-1	Partial response	67	Male	3	0	1	4
III-2	Stable disease	45	Female	2	0	0	2
III-3	Partial response	48	Female	3	0	0	3
III-4	Progressive disease	65	Female	1	0	1	4
III-5	Progressive disease	59	Male	3	1	1	4
III-6	Progressive disease	53	Female	4	0	0	3
III-7	Progressive disease	58	Male	1	0	0	1
III-8	Progressive disease	51	Male	3b	2	1	4
III-9	Partial response	71	Female	4	1	0	3
III-10	Stable disease	59	Male	1	0	0	1
III-11	Stable disease	70	Male	3	1	1	4
III-12	Partial response	47	Male	3	1	1	4

The study was conducted in accordance with the Declaration of Helsinki ethical principles. The patient groups, design of this study, and its public presentation in the form of a research paper were approved by the local ethical committees at I.M. Sechenov First Moscow State Medical University, Moscow City Oncological Hospital №. 62, and Moscow City Clinical Oncological Dispensary №. 1.

Biosamples were collected prospectively in the period from May 2015 until July 2020. All biosamples obtained from all the patients in this study were FFPE RCC tumor blocks obtained from primary tumor sites and evaluated by a pathologist, with cancer cell content of at least 60%. All patients were treated with sorafenib in first-line therapy and their responses were assessed according to RECIST criteria (Eisenhauer et al., 2009).

Twenty-two samples from kidney cancer patients were analyzed using Illumina HumanHT-12 WG-DASL V4.0 R2 gene expression array (Table 1). Among them, patients with progressive disease were considered as poor responders (*n* = 10), whereas patients with partial response and stable disease were classified as the responders (*n* = 12).

Gene expression for 13 other RCC samples was profiled by RNA sequencing using Illumina HiSeq3000 next generation sequencing platform (Table 2). Similarly, patients with progressive disease were considered as poor responders (*n* = 10), whereas patients with partial response and stable disease were classified as the responders (*n* = 3).

Finally, patients in the third set of 12 RCC patients were profiled by quantitative reverse transcription PCR (qRT-PCR) assay (Table 3). According to the aforementioned criteria, five patients were considered poor responders, and seven patients—treatment responders.

### Gene Expression Assays

#### RNA Extraction

To isolate RNA, 10-µm-thick paraffin slices were trimmed from each FFPE RCC tissue block using microtome. RNA was extracted from FFPE slices using QIAGEN RNeasy FFPE Kit following the manufacturer’s protocol. RNA 6000 Nano or Qubit RNA Assay kits were used to measure RNA concentration. RNA Integrity Number (RIN) was measured using Agilent 2100 bio-Analyzer.

#### Microarray Gene Expression Profiling

Gene expression profiling was done according to Lezhnina et al. (2014) at Dr. Olga Kovalchuk’s laboratory, University of Lethbridge, Alberta, Canada. The profiling was done using Illumina HumanHT-12 WG-DASL V4.0 R2 gene expression bead arrays. BeadChips were scanned using Illumina BeadArray Reader and the Bead Scan Software (Illumina).

#### RNA Sequencing

RNA sequencing was done according to Suntsova et al. (2019) and Sorokin et al. (2020d) at the Department of Pathology and Laboratory Medicine, University of California Los Angeles. For depletion of ribosomal RNA and library construction, KAPA RNA Hyper with rRNA erase kit (HMR only) was used. Different adaptors were used for multiplexing samples in one sequencing run. Library concentrations and quality were measured using Qubit ds DNA HS Assay kit (Life Technologies) and Agilent Tapestation (Agilent). RNA sequencing was done using Illumina HiSeq 3000 equipment for single-end sequencing, 50 bp read length, for approximately 30 million (mln) raw reads per sample. Data quality check was done on Illumina SAV. De-multiplexing was performed with Illumina Bcl2fastq2 v 2.17 program.

#### Quantitative Reverse Transcription PCR

Quantitative reverse transcription PCR (qRT-PCR) panel was developed to measure the expression level of eight target and six housekeeping genes in kidney cancer samples using Evrogen Reverse transcription polymerase and Evrogen Taq polymerase kit with SYBR Green for the PCR product detection. PCR mix composition included (25 µl total volume)

Buffer (HS-qPCRmix-HS SYBR; Evrogen, Moscow, Russia)—5 μl;
Primers 1 µl (0.4 µM each);
RNA solution—1–3 µl (2–6 ng total RNA per mix);
MMLV-RT (Evrogen)—2 µl;
Water—13–15 µl.

The oligonucleotide sequences for PCR primers are listed in Table 4. Following reverse transcription reaction, the PCR mix was melted at 95°C for 5 min, and then the following cycling conditions were applied using CFX Touch Real-Time PCR Detection System (BioRad):

95°C—30 s.
60°C—30 s.
72°C—30 s.

**TABLE 4:** Sequences of qRT-PCR primers used in this study

Target gene	Oligonucleotide sequence^a^ (5′–3′)
*RAF1*	F, *CTG​GCT​CCC​TCA​GGT​TTA​AGA​A*
	R, *AAG​CTC​CCT​GTA​TGT​GCT​CC*
*FLT3*	F, *CTC​AAG​GAA​ACG​GCC​ATC​CT*
	R, *AAC​ACG​GCC​ATC​CAC​ATT​CT*
*FLT1*	F, *TGT​CGT​GTA​AGG​AGT​GGA​CC*
	R, *GCA​CCT​GCT​GTT​TTC​GAT​GT*
*FGFR1*	F, *GAG​TGA​CTT​CCA​CAG​CCA​GA*
	R, *GGA​TGC​ACT​GGA​GTC​AGC​AG*
*BRAF*	F, *CAG​AGG​ACA​GTG​GTA​CCT​GC*
	R, *CAG​CAC​AGC​ACT​CTG​GGA​TT*
*PDGFRB*	F, *GCA​AAA​CCA​CCA​TTG​GGG​AC*
	R, *TGC​GTT​CAC​AGA​GAC​GTT​GA*
*KDR*	F, *GAA​ACT​GAC​TTG​GCC​TCG​GT*
	R, *CAC​GAC​TCC​ATG​TTG​GTC​ACT*
*KIT*	F, *GCA​CAA​TGG​CAC​GGT​TGA​AT*
	R, *GGT​GTG​GGG​ATG​GAT​TTG​CT*
*ACTB*	F, *ACAGAGCCTCGCCTTTGC*
	R, *CGC​GGC​GAT​ATC​ATC​ATC​CA*
*VCP*	F, *TGG​AAG​CGT​ATC​GAC​CCA​TC*
	R, *CTT​TGA​ACT​CCA​CAG​CAC​GC*
*DIABLO*	F, *AAT​GGC​GGC​TCT​GAA​GAG​TT*
	R, *AAA​CTC​GAG​CCA​AGC​AGG​AA*
*EIF3B*	F, *GGC​GAA​CAC​CAT​CTT​CTG​GA*
	R, *TGT​CCA​CAA​ACG​CTA​AGG​CA*
*PSMB2*	F, *GCA​GCA​GCT​AAC​TTC​ACA​CG*
	R, *AGC​CAG​GAG​GAG​GTT​CAC​AT*
*POLR2C*	F, *TCT​TCA​TCG​CTG​AGG​TTC​CC*
	R, *ATC​CAA​GCC​TGT​GAG​CAA​TGA*

a F—forward, R—reverse.

Each experiment was carried out in quadruplicate.

### Processing of Gene Expression Data

#### Illumina HumanHT-12 WG-DASL V4.0 R2 Gene Expression Array

Probe IDs were mapped to HGNC gene symbols (Yates et al., 2017) using the manufacturer’s annotation table. Gene expression values were normalized using quantile normalization protocol (Bolstad et al., 2003) prior to further processing. R package preprocessCore was used to perform quantile normalization.

#### Illumina HiSeq3000 RNAseq Profiles

RNA sequencing FASTQ files were processed with STAR aligner (Dobin et al., 2013) in “GeneCounts” mode with the Ensembl human transcriptome annotation (Build version GRCh38 and transcript annotation GRCh38.89). Ensembl gene IDs were converted to HGNC gene symbols using Complete HGNC dataset (https://www.genenames.org/, accessed on 2017 July 13). In total, expression levels were established for 36,596 annotated genes with the corresponding HGNC identifiers. Raw gene counts were normalized using R DESeq2 package (Love et al., 2014).

#### Quantitative Reverse Transcription PCR

For each sorafenib target gene from the RCC drug sensitivity gene signature (*RAF1*, *BRAF*, *FLT1*, *FGFR1*, *KIT*, *PDGFRB*, *FLT3*, *FLT4*, *KDR*), we performed normalization using expression of six housekeeping genes selected according to Chang et al. (2011) (*ACTB*, *GAPDH*, *POLR2C*, *PSMB2*, *DIABLO*, *VCP*). For each gene, we calculated ΔCt by subtracting the value of the threshold cycle of cDNA amplification of a target gene from the geometric mean value of the threshold cycle of cDNA amplification of the housekeeping genes. The gene signature score was calculated as sum of ΔCt values for all genes included in the signature.

### Gene Expression Analysis and Visualization

Differential gene expression analysis was performed using Student *t*-test. The observed clinical responses were used for investigation of molecular signature using ROC-AUC analysis (Fawcett, 2006). Area under a receiver operating characteristic curve (ROC-AUC) values were calculated using ROCR package in R environment (Sing et al., 2005). Patient survival analysis and visualization were performed using R packages *survival*, *survminer*, and *ggplot2*.

### Sorafenib *In Vitro* Efficiency Data

From Genomics of Drug Sensitivity in Cancer (GDSC) database (https://www.cancerrxgene.org/downloads/bulk_download, accessed on 2021 March 30), we downloaded log10-transformed IC_50_ values for sorafenib in 732 cancer cell lines corresponding to 13 different tumor types and 50 subtypes (Supplementary Table S1). For each cell line, we downloaded raw gene expression data from ArrayExpress database (https://www.ebi.ac.uk/arrayexpress/experiments/E-MTAB-3610/) in CEL format, experimentally profiled using Affymetrix Human Genome U219 Array. CEL files were normalized and background correction was applied using *rma* function of *affy* R package.

### Mutation Analysis

For mutation analysis, we used gene expression and genetic features data from GDSC database (https://www.cancerrxgene.org). We used data for 802 solid and 167 blood cancer cell lines with available genetic mutational profiles. For each mutation, we compared IC_50_ values for sorafenib between mutant and wild-type cell lines using non-parametric Mann–Whitney *U* test. Then we applied false discovery rate (FDR) correction to adjust for comparing multiple genetic features. Genetic features with FDR-corrected *p*-values <0.1 and more than 2-fold IC_50_ differences were considered as significant. Mann–Whitney *U* tests and FDR correction were performed using *scipy* and *statsmodels* Python libraries implemented in GDSC web interface.

## Results

### Study Population

In total, 47 RCC patients were enrolled in this study (21 female and 26 male patients, age range 41–74, mean 58 y.o.). The biosamples were FFPE RCC tumor tissue blocks collected in the period from May 2015 until July 2020. Gene expression was profiled using three different methods: microarray hybridization using Illumina HT-12 bead array, Illumina RNA sequencing, and qRT-PCR. Each patient provided a written informed consent and agreed that his/her biosample is profiled with one of the aforementioned methods. In the microarray group, there were 22 patients including 11 women and 11 men, age range 45–74, mean 59 y.o.; in RNAseq group—13 patients including 5 women and 8 men, age range 41–68, mean 56 y.o.; in qRT-PCR group, there were 12 patients including 7 men and 5 women, age range 45–71, mean 58 y.o. (Tables 1–3). The patients whose response status on next-line sorafenib monotherapy treatment according to RECIST criteria was “Progressive disease” were considered as poor responders, and the patients with statuses “Partial response” and “Stable disease” were considered as the responders. No “Complete response” outcomes according to RECIST (disappearance of all target lesions) were detected. This is in line with a previous study by Escudier et al., where only 1 out of 451 RCC patients treated with sorafenib had a complete response (Escudier et al., 2007). In total, 25 patients were classified as the poor responders, and 22—as the responders (Tables 1–3).

Specifically, there were 10 non-responders and 12 responders in the microarray group, 10 non-responders and three responders in the RNAseq group, and five non-responders and seven responders in the qRT-PCR group.

### Differential Gene Expression Analysis and Generation of Sorafenib Response Signature

**FIGURE 1:**
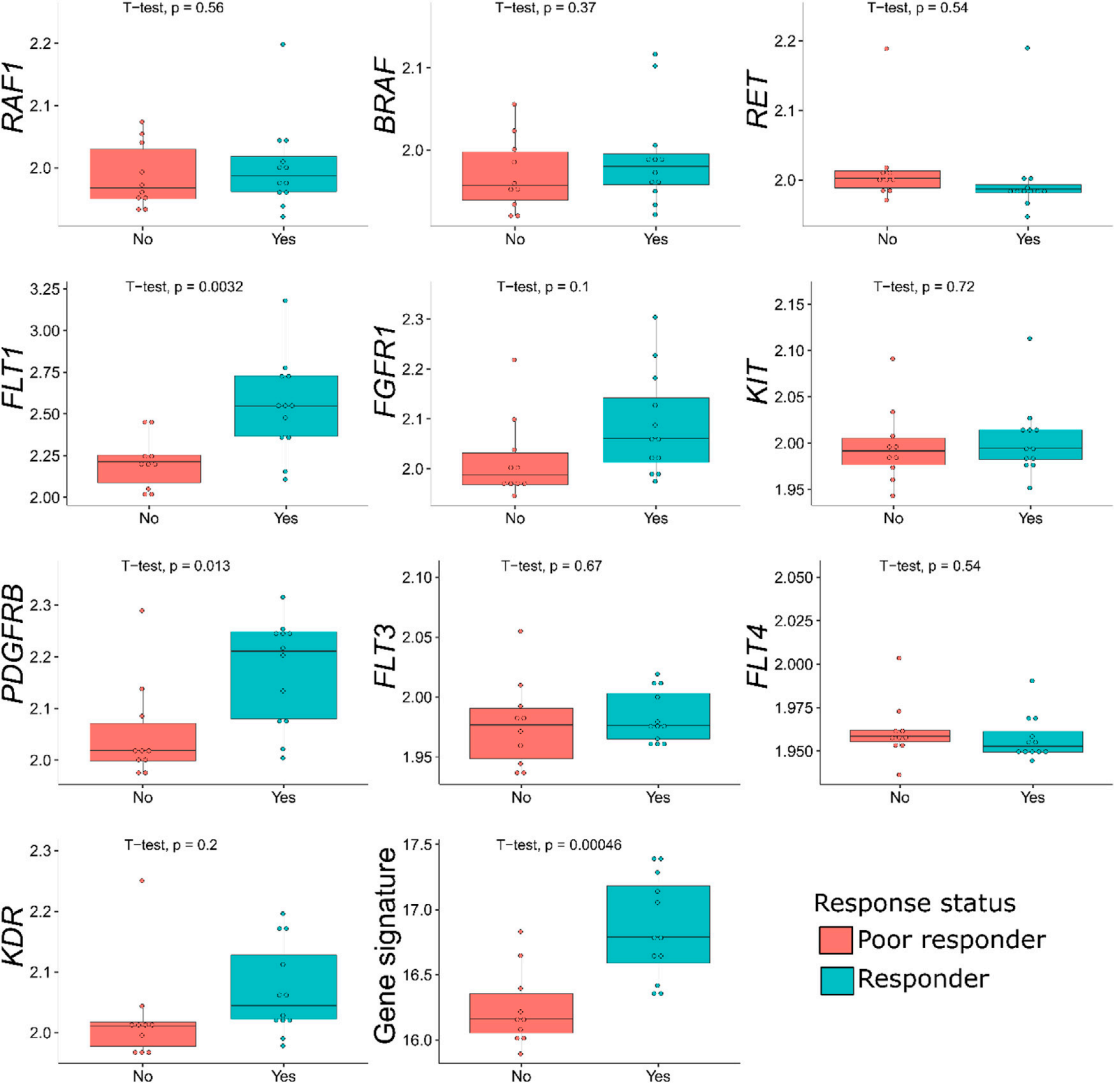
Distribution of sorafenib target gene expressions, and of the gene signature generated, among the sorafenib responder and poor responder groups of 22 RCC samples profiled by microarrays. For every gene, log10-transformed normalized expression is shown.

In the samples profiled by Illumina microarrays, we screened differential gene expression between the responder and poor responder biosamples. We aimed to generate sorafenib response gene signature and focused on expression levels of sorafenib target genes to avoid over-training. Using prior knowledge such as biological function of the genes is a well-established technique for feature selection as reviewed in Hira and Gillies (2015). At the single gene level, we observed a significant difference between the responders and poor responders only for *FLT1* and *PDGFRB* genes, which were both upregulated in the responders group (Table 5; Figure 1). Multiple logistic regression analysis did not provide significant coefficients for any of the sorafenib target genes.

**TABLE 5:** Differential expression analysis of sorafenib responders (*n* = 12) and poor responders (*n* = 10) in microarray-profiled RCC samples

HGNC gene ID	*T*-test *p*-value	Log_2_(fold change responders *vs.* poor responders)
*RAF1*	0.56	0.072
*BRAF*	0.37	0.071
*RET*	0.54	−0.037
*FLT1*	0.0032*	1.155
*FGFR1*	0.1	0.149
*KIT*	0.72	0.029
*PDGFRB*	0.013	0.273
*FLT3*	0.67	0.011
*FLT4*	0.54	−0.010
*KDR*	0.2	0.119

*p < 0.05.

In the previous studies, drug response statuses could correlate with the drug target gene expression levels (Tkachev et al., 2020), and for generating sorafenib drug response signature, we selected sorafenib target genes whose expression levels were greater in the responders than in the poor responders. Except two genes that were downregulated in the responders (*RET* and *FLT4*), the remaining eight sorafenib target genes that were upregulated were used to construct the molecular signature. Complex models with relatively small number of samples are often overfitted; therefore, we calculated the signature score as sum of log10-transformed normalized gene expression values, thus reducing data dimensionality.

This signature was tested to predict sorafenib response status in the microarray-profiled dataset. To assess the signature biomarker quality, we used AUC value as the measure. AUC reflects biomarker robustness and depends on its sensitivity and specificity (Borisov et al., 2020)⁠. It varies between 0.5 and 1, and the typical discrimination threshold is 0.7, where greater values denote high-quality biomarkers, and *vice versa* (Boyd, 1997)⁠. AUC is often used for scoring different types of molecular biomarkers in oncology (Liu et al., 2018; Tanioka et al., 2018; Chen et al., 2019; Sorokin et al., 2020a)⁠.

**FIGURE 2:**
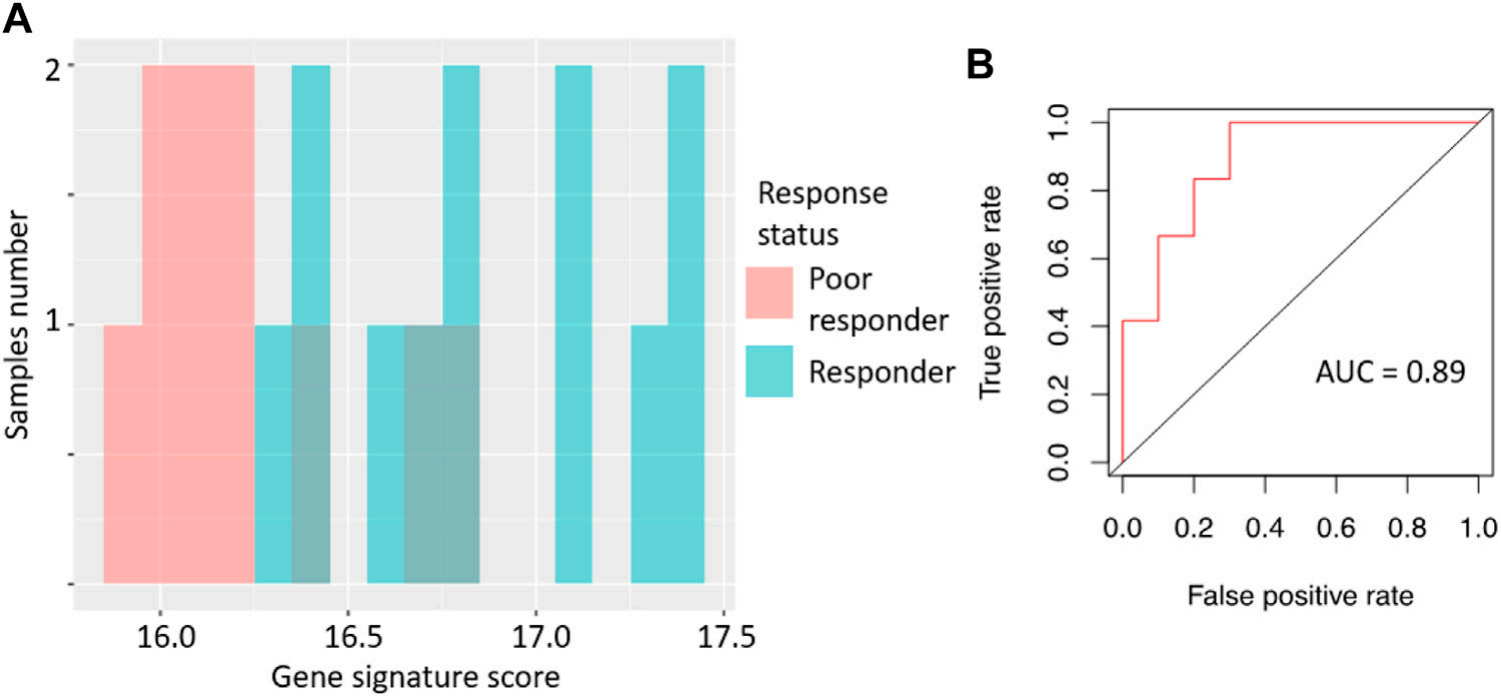
Performance of sorafenib response gene signature in microarray-profiled RCC set. **(A)** Distribution of gene signature score in 22 RCC samples profiled by expression microarrays. **(B)** ROC (receiver operating characteristic) curve for prediction of response status by gene signature score in 22 RCC samples profiled by expression microarrays. Validation of sorafenib response gene signature.

For the gene signature biomarker capacity, we obtained AUC value 0.89 (Figure 2B), which evidences its high prediction robustness. Using an assumption of equal frequency of type I and type II errors, we obtained threshold gene signature score of 16.41. This threshold corresponded to sensitivity 0.83, specificity 0.8, and Matthew’s correlation coefficient (MCC) 0.63; error matrix is shown on Supplementary Table S2. Interestingly, *t*-test *p*-value of the gene signature for comparison between the good and poor sorafenib responders (*p* = 0.00046) was lower than the respective *p*-value for any of the single sorafenib target genes (Figure 1).

We then tested the ability of the sorafenib response gene signature to predict good/poor response status using two alternative experimental platforms (RNAseq and qRT-PCR) and different sets of annotated RCC biosamples (*n* = 13 and *n* = 12, respectively).

**FIGURE 3:**
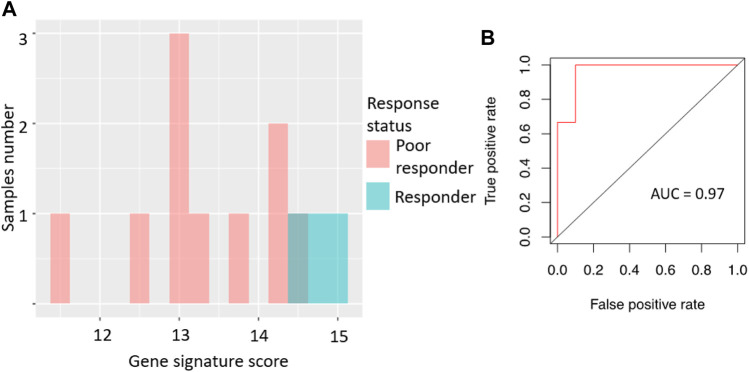
Performance of sorafenib response gene signature in RNAseq-profiled RCC set. **(A)** Distribution of gene signature score in 13 RCC samples profiled by RNA sequencing. **(B)** ROC (receiver operating characteristic) curve for prediction of response status by gene signature score in 13 RCC samples profiled by RNA sequencing.

**FIGURE 4:**
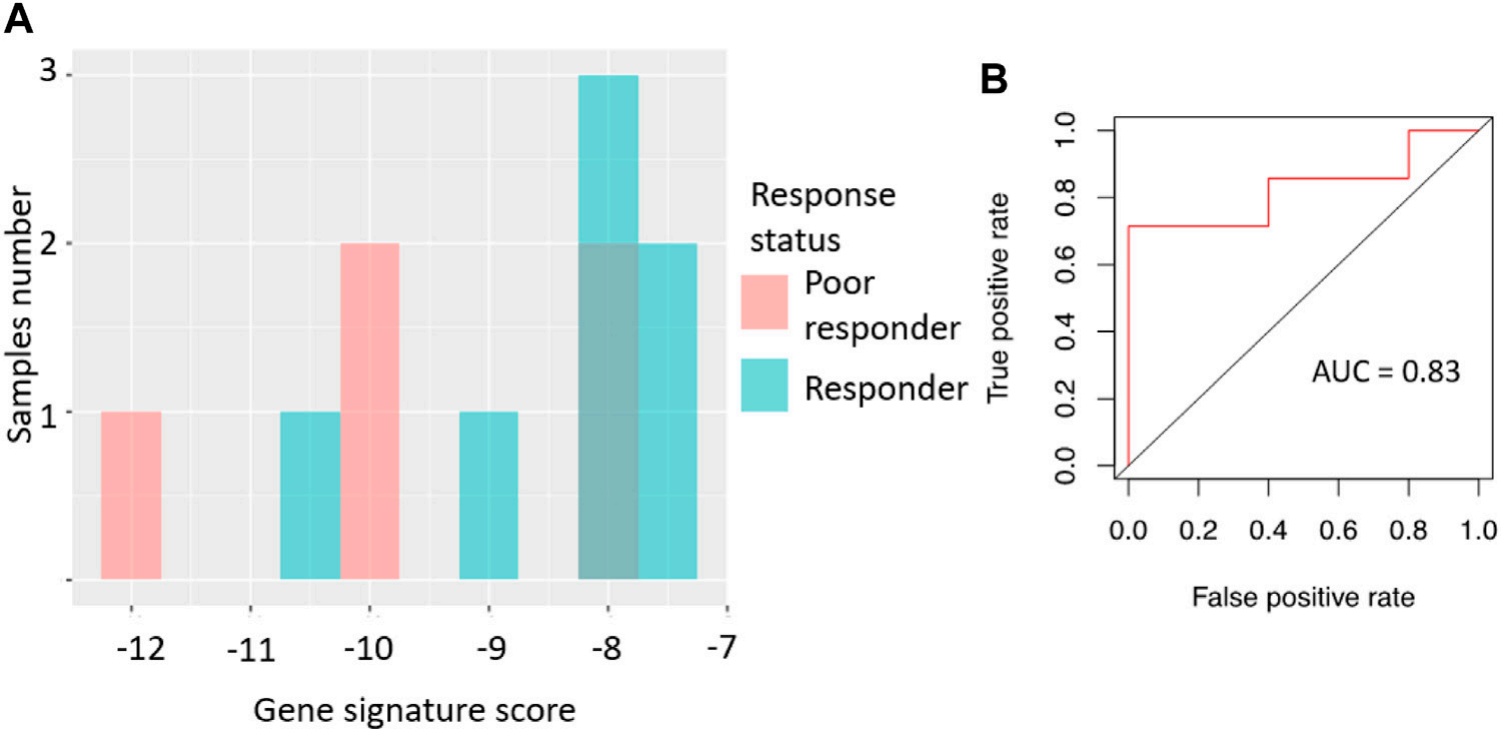
Performance of sorafenib response gene signature in microarray-profiled RCC set. **(A)** Distribution of gene signature score in 22 RCC samples profiled by expression microarrays. **(B)** ROC (receiver-operator characteristic) curve for prediction of response status by gene signature score in 22 RCC samples profiled by expression microarrays. *In vitro* validation of sorafenib response gene signature.

Specifically, the signature score for RNAseq data was calculated in the same way as for the microarray dataset: sum of the log-transformed expression values for the same eight sorafenib target genes. For the qRT-PCR dataset, we totalized ΔCt values for the selected sorafenib targets. For those two platforms, we obtained AUC scores of 0.97 and 0.83, respectively (Figures 3, 4; error matrices are shown on Supplementary Tables S3, S4, respectively).

For the RNAseq dataset, we also used an assumption of equal frequency of type I and type II errors and obtained threshold gene signature score of 14.35, sensitivity 1.00, specificity 0.9, and MCC 0.82. Similarly, in the case of qRT-PCR dataset, a threshold of −8.03 was obtained, which corresponded to sensitivity 0.71, specificity 1.0, and MCC 0.71.

The high scores of AUC, MCC, sensitivity, and specificity values observed for all three cohorts suggest in favor of the proposed sorafenib response gene signature usefulness as the new combinatorial expression biomarker.

We further validated the sorafenib response gene signature using bioinformatics analysis of publicly available cell line gene expression data annotated with sorafenib sensitivity information. We calculated molecular signature scores for 735 samples of different cancer cell lines extracted through the GDSC database (Yang et al., 2013).

**FIGURE 5:**
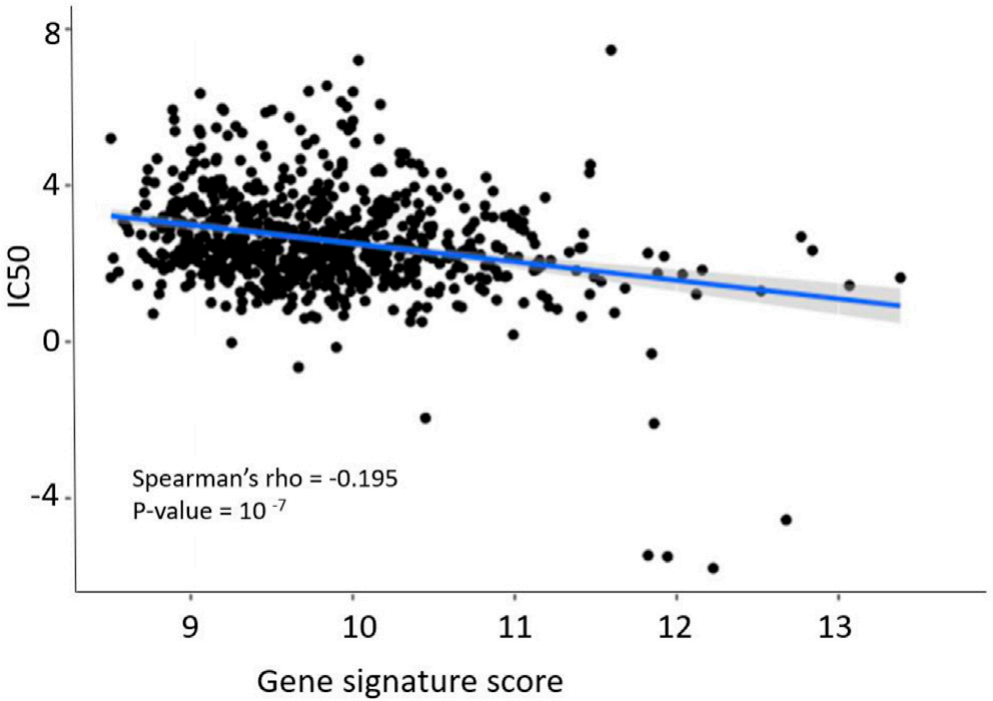
Dependence of sorafenib resistance gene signature score and sorafenib IC_50_ in GDSC pan-cancer dataset. Blue line and shadow around it show linear approximation and 5% confidence interval. Figure built using *ggplot* function in R.

We then compared gene signature scores with the log10-transformed IC_50_ micromolar values of sorafenib. IC_50_ shows sorafenib concentration that reduces cell viability by 50%, and therefore IC_50_ is an inverse measure of drug efficiency (high IC_50_ suggests strong drug resistance, and low IC_50_ means high sensitivity to a drug). We observed a statistically significant negative correlation between sorafenib IC_50_ and gene signature score (Figure 5), Spearman correlation −0.195, *p* = 10^−7^.

**FIGURE 6:**
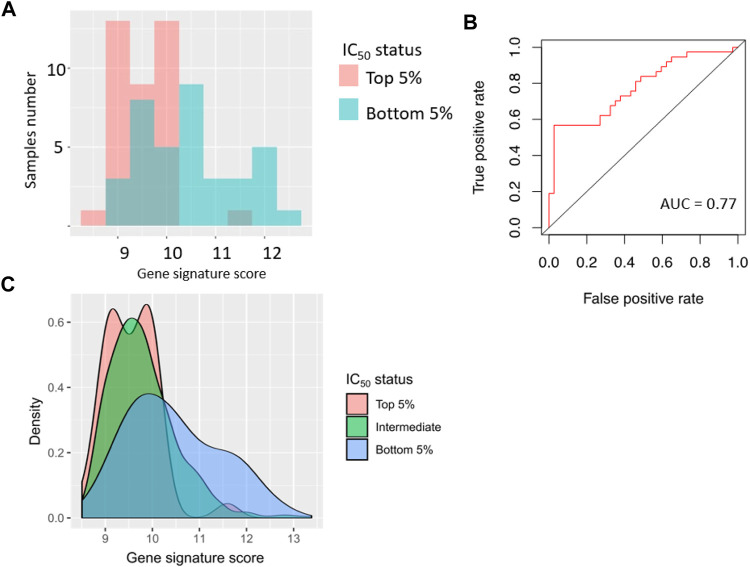
Performance of sorafenib response gene signature in 76 cancer cell lines (top 5% and bottom 5% cell lines from GDSC dataset, sorted by sorafenib IC_50_). **(A)** Distribution of gene signature score in 76 cancer cell lines. **(B)** ROC (receiver operating characteristic) curve for prediction of response status by gene signature score in 76 cancer cell lines. **(C)** Distribution of gene signature score in all cancer cell lines.

We then modeled ability of the gene signature to predict sorafenib IC_50_ in the tumor cell lines. Using GDSC data, we selected top and bottom 5% cell lines by sorafenib IC_50_, and associated them with the sorafenib poor and good responders, respectively. In this test, AUC value for prediction of high or low sorafenib IC_50_ by gene signature score was 0.77 (Figure 6).

Based on the assumption of equal importance of type I and type II errors, in this setting we obtained gene signature score threshold of 9.8, MCC coefficient 0.32, sensitivity 0.63, and specificity 0.66; error matrix for this analysis is shown on Supplementary Table S5.

### Mutations Associated With Sorafenib Activity *In Vitro*

**FIGURE 7:**
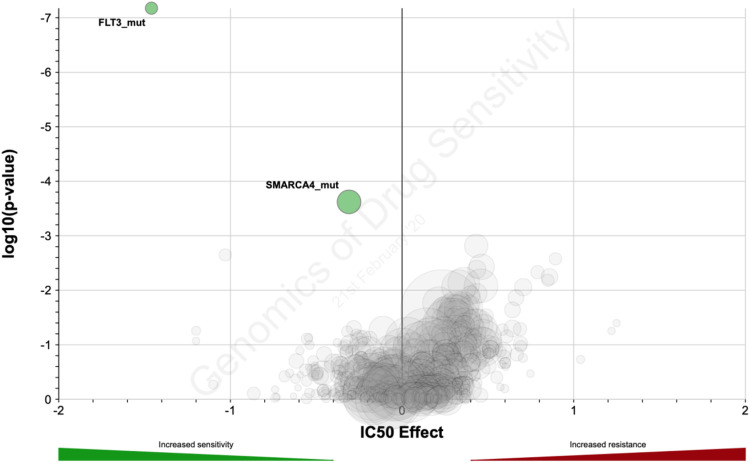
Distribution of log10-transformed *p*-value and IC_50_ difference between groups with and without gene-specific mutations and copy number alternations in GDSC database.

Using the GDSC dataset, we further investigated the connection between sorafenib IC_50_ and annotated mutations in the GDSC cell lines. *P*-value cut-off was set according to GDSC default parameters (threshold FDR corrected *p* < 0.1 and fold change >2). With the internal GDCS analytic interface, we identified mutations in two genes that were statistically significantly linked with IC_50_ of sorafenib: *FLT3* and *SMARCA4* (Figure 7; Table 6). The observed genetic features contained different driver mutations in both genes. Noteworthy, *FLT3* gene product is one of the molecular targets of sorafenib. Thus, strong linkage of driver mutations in this gene with the sensitivity to sorafenib directly confirms its implication in the mechanisms of cancer cells’ resistance to sorafenib. For the second gene (*SMARCA4*), we found no previous associations with sorafenib efficacy in the literature. However, molecular function of this gene product is ATP-dependent chromatin remodeling and overall transcriptional activation, and *SMARCA4* mutations are linked with many cancers (Fountzilas et al., 2021; Nambirajan and Jain, 2021; Pastorczak et al., 2021).

**TABLE 6:** Genes which mutations are statistically significantly associated with sorafenib IC_50_ in GDSC data collection

Gene mutation	IC_50_ effect size (fold change)	*p*-value	FDR	Number of altered cell lines
*FLT3*_mut	−1.46	6.67 × 10^−08^	0.000042	11 (1.6%)
*SMARCA4*_mut	−0.309	0.000239	0.0754	41 (6%)

## Discussion

Sorafenib is a targeted tyrosine kinase inhibitor (TKI) with multiple molecular specificities, which is widely used to treat kidney cancer due to relative clinical efficacy and affordability (Sheng et al., 2016; Cai et al., 2017). However, sorafenib response rate in RCC varies between 10 and 40% (Larkin and Eisen, 2006; Escudier et al., 2007; Guevremont et al., 2009), thus personalized approach is needed to select the patients who would more likely benefit from the treatment with this drug.

High-throughput gene expression profiling is becoming a powerful tool for finding new cancer biomarkers (Buzdin et al., 2019; Tsimberidou et al., 2020). Moreover, aggregating gene expression levels into functional groups like molecular pathways or gene signatures can increase efficiency of the biomarkers and even enhance stability of experimental data (Borisov et al., 2017; Buzdin et al., 2018). Previously we used this approach to establish biomarkers of trastuzumab response in metastatic/recurrent HER2-positive breast cancers (Sorokin et al., 2020a), ramucirumab response in gastric cancer (Sorokin et al., 2020d), and for building gene signature for ganglioside GD2 expression in cancer cells (Sorokin et al., 2020c).

In this study, we identified and validated an 8-gene expression signature that predicts sorafenib response in RCC patients. The signature was validated on the independent patient groups using three different methods of gene expression profiling: by Illumina HT-12 microarrays, by RNA sequencing, and by qRT-PCR. The sorafenib response signature includes eight sorafenib target genes: *RAF1*, *BRAF*, *FLT1*, *FGFR1*, *KIT*, *PDGFRB*, *FLT3*, and *KDR*. Among them, increased expressions of single genes *FLT1* and *PDGFRB* were positively associated with the sorafenib response, whereas other genes showed similar trends, which were however not statistically significant. At the same time, the gene signature could show better efficacy than any of the separately taken enclosing genes, thus evidencing better efficacy of a cumulative complex expression biomarker. On the other hand, significant association of sorafenib target gene *FLT3* was confirmed at the level of driver mutations in GDSC data, thus implying a peculiar role for this gene in the sorafenib activity mechanism.

Sorafenib has a strong overlap in the molecular specificities with regorafenib (Granito et al., 2021) and with several other TKI drugs (Shah et al., 2020; Das et al., 2021), and theoretically the same drug target–based gene signature approach can be translated on finding new response biomarkers for other TKIs as well, and for different cancer types. However, such an approach would require accumulating enough tumor gene expression data connected with the specific drug response statuses, which is frequently a difficult task to implement. For example, to the best of our knowledge, the high-throughput experimental expression profiles that were associated here with the sorafenib response are the first such RCC dataset published in the literature. Accumulation and publishing of more molecular profiles connected with the TKI response statuses in RCC and other tumors would clearly enhance development of next-generation drug response prediction biomarkers.

For the current sorafenib 8-gene expression signature, we developed a qRT-PCR–based diagnostic panel that enables cost-effective molecular profiling. The panel was validated on an independent cohort of RCC patients with AUC = 0.83, which opens an avenue for further molecular testing on bigger patient cohorts and, if successful, for the development of diagnostic tools supporting personalized sorafenib prescriptions. Such a study would also be needed to validate the exact threshold developed for qRT-PCR signature established herein.

Interestingly, the same 8-gene signature was also validated using GDSC project cell line gene expression data connected with the tested drug sensitivities (Yang et al., 2013): a modest (−0.195) yet highly statistically significant (*p* = 10^−7^) correlation was observed for the gene signature score and sorafenib IC_50_. The GDSC collection accumulated data for various cancer cell lines. Cell lines are heterogeneous and derived from tumors of various origin, not only kidney cancer. In addition, *in vitro* culturing may have an impact on gene expression. Despite all these factors, we still obtained statistically significant performance of the gene signature. Potentially, this may indicate that this gene signature is not specific to RCC but may be also predictive for the other cancer types. Thus, further clinical investigations are needed to assess its performance in cancers other than RCC.

## Data Availability

The datasets presented in this study can be found in online repositories. The names of the repository/repositories and accession number(s) can be found below: https://www.ncbi.nlm.nih.gov/bioproject/PRJNA749745/, https://www.ncbi.nlm.nih.gov/geo/, GSE180925.
